# Fulminant neonatal listeriosis with death within 24 hours in a preterm infant: a case report

**DOI:** 10.1186/s12879-025-11641-8

**Published:** 2025-10-06

**Authors:** Xiaohong Tao, Haiting Li, Jie Li, Xiyang Chen, Linlin Chen, Junhui Yuan, Enfu Tao, Xixi Liu

**Affiliations:** 1 Department of Pediatrics, Wenling Maternal and Child Health Care Hospital, Wenling, Zhejiang Province China; 2 Department of Neonatology and Neonatal Intensive Care Unit, Wenling Maternal and Child Health Care Hospital, 102 Xiabao Road, Chengdong Street, Wenling, Zhejiang Province 317500 China

**Keywords:** *Listeria monocytogenes*, Neonatal sepsis, Septic shock, Preterm infant, Foodborne infection

## Abstract

**Background:**

Neonatal listeriosis, caused by *Listeria monocytogenes*, represents a rare yet severe infection that can result in fatal outcomes, especially among preterm infants; however, instances of mortality occurring within 24 h of diagnosis are exceedingly uncommon.

**Case presentation:**

This report presents the case of a preterm female infant who developed fulminant listeriosis with rapid progression to septic shock and multiorgan failure. The patient was born at 33 5/7 weeks, weighed 2,395 g, and presented with severe respiratory distress and perinatal asphyxia. The mother had consumed refrigerated leftovers and developed fever (38.5 °C) one day prior to delivery. Upon admission, the infant exhibited metabolic acidosis (pH 7.14, lactate 10 mmol/L), leukopenia (2.7 × 10⁹/L), and septic shock. Despite immediate mechanical ventilation, inotropic support, and broad-spectrum antibiotics (penicillin and meropenem), the infant succumbed within 24 h. Blood and cerebrospinal fluid cultures confirmed *Listeria monocytogenes* infection.

**Conclusions:**

This case emphasizes the deadly risk of neonatal listeriosis and the need for preventive measures, such as dietary precautions for pregnant women (avoiding unpasteurized dairy and properly stored leftovers) and early maternal diagnosis and treatment. Prompt recognition and antibiotic therapy for maternal listeriosis are crucial for better outcomes in this vulnerable group.

## Introduction

*Listeria monocytogenes* is recognized as a significant pathogen, particularly affecting vulnerable populations such as neonates, pregnant women, and individuals with compromised immune systems [1, 2]. Although infections caused by *Listeria* are relatively uncommon in healthy children, the risk is considerably elevated in preterm infants due to their immature immune systems [1, 3]. Neonatal listeriosis can manifest with a spectrum of clinical symptoms, including erythematous rash, intractable convulsions, and severe early-onset neonatal sepsis, which may rapidly progress to disseminated intravascular coagulation and multiple organ dysfunction syndrome [4, 5]. These severe complications can result in fatal outcomes, even with intensive therapeutic interventions [4].

The primary route of neonatal *Listeria* infection is transplacental transmission from the mother, often linked to maternal ingestion of contaminated food [2, 6]. In cases where maternal listeriosis is not promptly treated, the neonatal mortality rate is alarmingly high, potentially reaching up to 50% [7]. Consequently, for pregnant women with suspected *Listeria* infection, timely administration of antibiotic therapy is essential to mitigate the severity of neonatal *Listeria* infection and enhance prognosis [4]. Additionally, due to the aggressive nature of this foodborne illness, it is imperative for expectant mothers to refrain from consuming contaminated food.

We present a case of *Listeria* infection in a preterm neonate (gestational age 33 5/7 weeks) following maternal ingestion of refrigerated leftovers during late pregnancy. Shortly after birth, the neonate’s condition deteriorated rapidly, leading to septic shock, capillary leak syndrome (CLS), and multiorgan failure within 24 h, ultimately resulting in fatality.

## Case presentation

A female infant, born preterm at 33 5/7 weeks of gestation in mid-March (spring season) weighing 2395 g, was admitted to our neonatal intensive care unit due to perinatal asphyxia and severe respiratory distress. The infant was delivered vaginally in the presence of meconium-stained amniotic fluid, characterized by third-degree turbidity and a volume of 150 ml. There was no clinical evidence of preeclampsia, preterm premature rupture of membranes, placental abruption, or cervical insufficiency. The newborn exhibited non-vigorous status at delivery, prompting immediate resuscitation measures including meconium suctioning and thorough airway clearance. Due to persistent bradycardia and inadequate respiratory effort despite 30 s of effective positive-pressure ventilation, the resuscitation team initiated chest compressions. The infant’s clinical status showed gradual improvement, with Apgar scores documenting 3 at 1 min and 6 at 5 min post-delivery. Subsequently, she was transferred to our neonatal unit. Two weeks prior to delivery, the mother developed a cough and sputum production, which remained untreated. One day before delivery, the mother experienced a fever, with a peak temperature of 38.5 °C, along with persistent cough and sputum production, for which she self-administered oral ibuprofen liquid. Her fever persisted, and at the time of delivery, her temperature had risen to 38.8 °C. Additionally, despite having received prenatal advice against consuming high-risk foods, the mother reported consuming store-bought milk and home-cooked seafood that had been refrigerated overnight after being left at room temperature for approximately four hours (storage temperature: 8 °C).

**Fig. 1:**
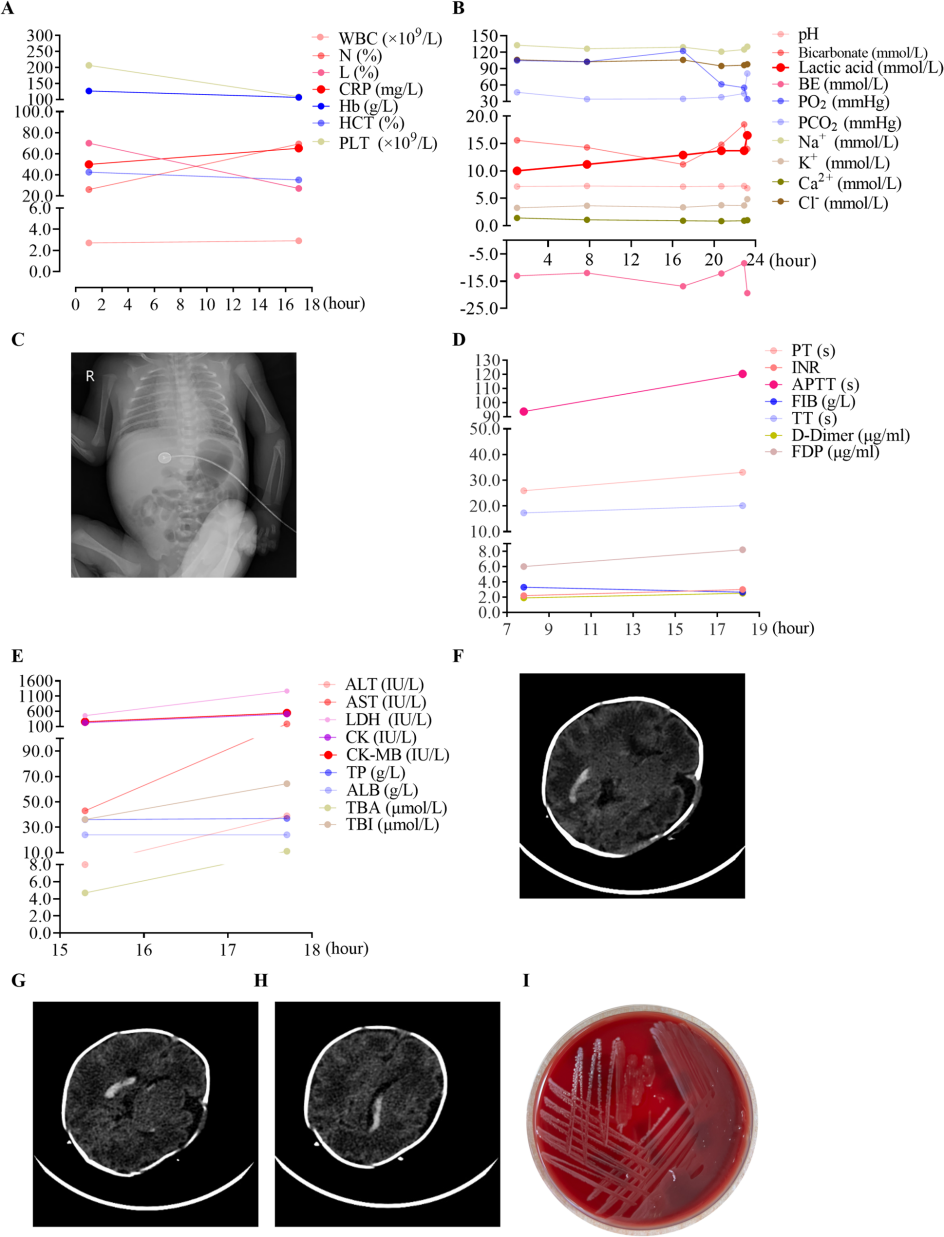
Dynamic changes in representative laboratory and imaging findings. A: complete blood count, B: blood gas, C: chest X-ray, D: cougulation function, E: blood biochemistry, F, G, H: cranial computed tomography scan, I: blood culture. WBC: white blood cell, RBC: red blood cell, Hb: hemoglobin, HCT: hematocrit, PLT: platelet count, N: neutrophil, CRP: C-reactive protein, ALT: alanine aminotransferase, AST: aspartate aminotransferase, TBI: total bilirubin; TBA: total bile acid, TP: total protein, ALB: Albumin, LDH: lactate dehydrogenase; CK: creatine kinase, CK-MB: creatine kinase-MB isoenzyme. pH: potential of hydrogen, PO_2_: partial pressure of oxygen, PCO_2_: partial pressure of carbon dioxide, BE: base excess.

Upon admission, the patient’s vital signs were recorded as follows: a temperature of 36.8 °C, a heart rate of 170 beats per minute, a respiratory rate of 66 breaths per minute, and a blood pressure of 45/22 mmHg. The physical examination indicated poor responsiveness, a weak cry, tachypnea accompanied by grunting, hypotonia, and mottled skin on the extremities. Initial laboratory investigations revealed a blood glucose level of 2.7 mmol/L, while blood gas analysis indicated metabolic acidosis, with a pH of 7.14, a base excess of −13.04 mmol/L, and a lactate level of 10.0 mmol/L (Fig. 1A). A complete blood count demonstrated leukopenia (2.7 × 10⁹/L), lymphocytosis (70.1%), and an elevated C-reactive protein level (50.0 mg/L) (Fig. 1B). A chest X-ray exhibited enhanced and disordered lung markings with blurred edges, as well as multiple scattered patchy and punctate opacities throughout both lung fields (Fig. 1C). Additionally, an abdominal ultrasound revealed the presence of ascites.

The infant was administered mechanical ventilation for respiratory support, along with inotropic therapy utilizing dopamine and dobutamine to ensure hemodynamic stability. Broad-spectrum antimicrobial therapy was initiated with a combination of penicillin and cefoperazone/sulbactam, which was subsequently escalated to penicillin and meropenem due to the persistence of sepsis. Additionally, antiviral therapy with acyclovir was provided to address potential viral involvement. Supportive interventions included blood transfusions, plasma infusions, and other adjunctive measures aimed at managing coagulopathy and hemodynamic instability. Despite the implementation of aggressive treatment strategies, the patient’s condition deteriorated rapidly, leading to refractory shock characterized by persistent hypotension, further complicated by capillary leak syndrome (CLS), which manifested as generalized edema, hyponatremia, and hypocalcemia. The clinical picture was further exacerbated by worsening metabolic acidosis, coagulopathy, and multiorgan failure, alongside a progressive decline in laboratory parameters (Fig. 1A, B, D, E). Cranial ultrasound findings indicated bilateral subependymal hemorrhage, while cranial computed tomography revealed intraventricular hemorrhage (IVH) and subarachnoid hemorrhage (SAH) (Fig. 1F, G, H). Echocardiographic assessment revealed the presence of a patent ductus arteriosus (PDA) (diameter 3.0 mm) and a patent foramen ovale (PFO) (diameter 2.4 mm), accompanied by tricuspid regurgitation. Despite extensive medical interventions, the infant unfortunately succumbed to her illness within 24 h of admission. Blood and cerebrospinal fluid (CSF) cultures confirmed an infection with *Listeria monocytogenes* 48 h post-admission (Fig. 1).

The final diagnosis included neonatal sepsis, septic shock, CLS, multiple organ dysfunction syndrome, neonatal respiratory distress syndrome (RDS), severe neonatal asphyxia, neonatal meconium aspiration syndrome (MAS), metabolic acidosis, neonatal anemia, neonatal intracranial hemorrhage, neonatal hypoxic-ischemic encephalopathy (HIE), low birth weight infant, appropriate-for-gestational-age (AGA), neonatal hyponatremia, neonatal hypocalcemia, PFO, PDA, and ascites. The infant’s death resulted from multiorgan failure secondary to *Listeria monocytogenes* sepsis and septic shock, exacerbated by severe neonatal asphyxia and complications of prematurity. The mother’s postpartum course was uncomplicated. Her temperature normalized promptly after delivery, and she exhibited no clinical or histopathological evidence of chorioamnionitis. She was discharged home on postoperative day three after both blood and cervical swab cultures returned negative.

## Discussion

This report describes a fatal case of fulminant neonatal listeriosis in a preterm infant with rapid disease progression leading to death within 24 h of birth, resulting from transplacental transmission following maternal consumption of contaminated refrigerated food. This tragic outcome highlights the exceptionally aggressive nature of perinatal listeriosis and underscores the crucial importance of strict dietary precautions during pregnancy, particularly the avoidance of potentially contaminated food products.

Neonatal listeriosis, a severe foodborne infection caused by *Listeria monocytogenes* [8], has an incidence of 2.06 per 100,000 live births in China, with 73% of cases occurring in preterm infants, typically presenting as early-onset sepsis (EOS) [9]. This condition exhibits a seasonal predominance during the spring and summer months, with affected mothers commonly reporting symptoms such as fever, abdominal pain, and vaginal discharge, while neonates primarily exhibit respiratory distress and fever [10]. The infection can manifest as sepsis and central nervous system involvement, associated with alarmingly high mortality rates ranging from 20 to 30% [8, 9, 11]. The causative organism, a Gram-positive facultative Anaerobe, demonstrates remarkable environmental adaptability, surviving temperatures from 1 °C to 45 °C (including refrigeration) and exhibiting resistance to desiccation, high salinity, and acidic conditions. This adaptability renders it a particularly hazardous pathogen for vulnerable populations, including pregnant women, newborns, and immunocompromised individuals [8].

Premature infants infected with *Listeria monocytogenes* are at risk of rapid clinical decline, leading to fatal outcomes within short timeframes. In the present case, the neonate succumbed within merely 24 h post-delivery, with this fulminant progression attributable to multiple pathogenic mechanisms. First, as a Gram-positive facultative intracellular pathogen, *Listeria monocytogenes* can invade and survive within host cells, including macrophages and epithelial cells [12]. This capability enables the bacterium to evade host immune surveillance and proliferate within the host’s cellular environment, particularly in premature infants with underdeveloped immune systems [13]. The bacterium employs virulence factors such as listeriolysin O (LLO) to disrupt host cell membranes, facilitating its escape from phagosomes and promoting intracellular survival and dissemination. LLO, a pore-forming toxin, creates openings in the phagosomal membrane, allowing the bacteria to escape into the cytoplasm where they can multiply and spread to adjacent cells [14]. Second, the immature immune system of preterm neonates is characterized by inadequate defense mechanisms, which renders these individuals particularly susceptible to infections [12]. *Listeria monocytogenes* has developed various strategies to evade host immune responses. The bacterium is capable of modulating inflammatory responses and suppressing cytokine production, thereby circumventing the host’s immune defenses. Furthermore, it can inhibit the activation of T cells and natural killer (NK) cells, which further undermines the host’s capacity to mount an effective immune response [13]. Additionally, infection with *Listeria monocytogenes* can induce mitochondrial dysfunction in host cells, resulting in compromised cellular homeostasis and impaired immune function [14]. This mitochondrial dysfunction may exacerbate the severity of the infection by weakening the host’s cellular defense mechanisms. Importantly, infection with *Listeria monocytogenes* can also lead to placental dysfunction through inflammatory changes and direct microbial invasion [15], which is a critical factor contributing to rapid clinical deterioration in neonates. The resultant placental inflammation significantly jeopardizes fetal viability. Finally, the pathogen’s capacity to disrupt host cell signaling pathways significantly accelerates disease progression by undermining immune defenses [16]. This disruption can result in unchecked bacterial proliferation and severe systemic infection. The rapid clinical deterioration observed in preterm neonates with listeriosis can be attributed to a synergistic interplay of several factors: (1) bacterial virulence mechanisms that facilitate systemic dissemination, (2) inherent developmental immune deficiencies, and (3) infection-induced placental and cellular dysfunction. Collectively, these elements contribute to the characteristic rapid onset of severe clinical manifestations and elevated mortality rates within this vulnerable population. This case study involves a preterm neonate (33 5/7 weeks) who experienced severe perinatal asphyxia, which likely exacerbated immunological compromise and physiological stress responses, potentially worsening the outcomes of *Listeria monocytogenes* infection [17]. The mother presented with prodromal symptoms, including cough and sputum production, two weeks prior to delivery and developed a fever (38.5 °C) one day before delivery, having consumed refrigerated leftovers—a recognized risk factor for *Listeria monocytogenes* infection—without receiving appropriate antibiotic therapy [18]. This resulted in significant transplacental bacterial transmission, leading to fulminant EOS characterized by rapid and fatal progression. In this instance, despite the prompt administration of penicillin-class antibiotics, the infection remained uncontrollable, ultimately culminating in fatal outcomes. While our case benefited from timely diagnosis and intervention, the irreversible severity of the outcome serves as a powerful reminder of the paramount importance of primary prevention through strict dietary avoidance during pregnancy [19].

As a disease associated with pregnancy, the prevention of *Listeria monocytogenes* infection is paramount in mitigating morbidity and mortality rates. It is essential to provide dietary guidance to pregnant women to enhance their awareness of food safety and assist them in avoiding the consumption of contaminated food [9]. This guidance should emphasize the avoidance of high-risk items, including unpasteurized dairy products, refrigerated ready-to-eat meats, and soft cheeses made from unpasteurized milk. Furthermore, adherence to proper food handling and storage practices—such as maintaining refrigerator temperatures below 4 °C and refraining from consuming leftovers stored for more than 24 h—is critical in reducing the risk of infection. Prenatal counseling and education aimed at increasing public awareness of foodborne disease risks are also vital components of listeriosis prevention during pregnancy [10]. Additionally, healthcare providers have a crucial role in educating high-risk populations, particularly pregnant women, about the dangers associated with the consumption of contaminated food and the significance of hygiene practices. By prioritizing these preventive measures, we can substantially decrease the incidence of listeriosis among vulnerable populations [18].

Additionally, despite this compelling exposure history and the subsequent confirmed infection in the neonate, all maternal microbiological cultures yielded negative results. This discrepancy underscores a key clinical challenge in perinatal listeriosis: the frequent absence of laboratory confirmation in the mother despite her being the likely source. The most plausible explanation for this discrepancy is that the mother experienced a transient, subclinical bacteremia. *Listeria monocytogenes* is characterized by its pronounced tropism for the fetoplacental unit and is often cleared from the maternal bloodstream by the time of delivery, accounting for the negative cultures [20]. This is corroborated by literature indicating that maternal bacteremia is not always detectable; for instance, one study reported positive blood cultures in only 55% of perinatal listeriosis cases [21]. Furthermore, the absence of chorioamnionitis, despite overwhelming fetal infection, highlights that a robust placental inflammatory response is not a prerequisite for microbial transmission. The reported incidence of chorioamnionitis in maternal listeriosis varies widely (ranging from 5.8% to 100% in different studies [22, 23]), indicating significant heterogeneity in the host inflammatory response. More importantly, the critical pathogenic step is the bacterial breaching of the trophoblast barrier [24]. This invasion can lead to rapid neontal infection without necessarily eliciting a massive neutrophilic infiltrate in the placenta [22], explaining the absence of chorioamnionitis in our case.

## Conclusion

This fatal case underscores the lethal potential of neonatal listeriosis in preterm infants, where rapid progression to septic shock and multiorgan failure can occur despite aggressive treatment. The findings emphasize the critical importance of maternal food safety education—particularly avoiding refrigerated leftovers and high-risk foods—to prevent transplacental transmission. Furthermore, in febrile pregnant women with a history of dietary exposure, a high index of suspicion for listeriosis should be maintained even in the absence of characteristic microbiological or pathological findings. Early recognition of maternal listeriosis and prompt antibiotic therapy remain essential to improving outcomes in this vulnerable population.

## Data Availability

The data used during the study are available from the corresponding author.

## Abbreviations

CLS: Capillary leak syndrome
IVH: Intraventricular hemorrhageand
SAH: Subarachnoid hemorrhage
CSF: Cerebrospinal fluid
RDS: Respiratory distress syndrome
MAS: Meconium aspiration syndrome
HIE: Hypoxic-ischemic encephalopathy
AGA: Appropriate-for-gestational-age
PFO: Patent foramen ovale
PDA: Patent ductus arteriosus
EOS: Early-onset sepsis
LLO: Listeriolysin O
NK: Natural killer
WBC: White blood cell
RBC: Red blood cell
Hb: Hemoglobin
HCT: Hematocrit
PLT: Platelet count
N: Neutrophil
CRP: C-reactive protein
ALT: Alanine aminotransferase
AST: Aspartate aminotransferase
TBI: Total bilirubin
TBA: Total bile acid
TP: Total protein
ALB: Albumin
LDH: Lactate dehydrogenase
CK: Creatine kinase
CK-MB: Creatine kinase-MB isoenzyme
pH: Potential of hydrogen
PO_2_: Partial pressure of oxygen
PCO_2_: Partial pressure of carbon dioxide
BE: Base excess
